# Rapid identification of the invasive fall armyworm *Spodoptera frugiperda* (Lepidoptera, Noctuidae) using species-specific primers in multiplex PCR

**DOI:** 10.1038/s41598-020-73786-7

**Published:** 2020-10-05

**Authors:** Cheng-Lung Tsai, I.-Hsuan Chu, Ming-Hsun Chou, Theeraphap Chareonviriyaphap, Ming-Yao Chiang, Po-An Lin, Kuang-Hui Lu, Wen-Bin Yeh

**Affiliations:** 1 Department of Entomology, National Chung Hsing University, 145 Xingda Rd., South District, Taichung City, 40227 Taiwan; 2 Department of Entomology, Faculty of Agriculture, Kasetsart University, 50 Ngamwongwan Rd., Chatuchak, Bangkok, 10900 Thailand; 3 Applied Zoology Division, Taiwan Agricultural Research Institute, Council of Agriculture, Executive Yuan, 189 Zhongzheng Rd., Wufeng District, Taichung, 41326 Taiwan; 4 Department of Entomology, Penn State University, 201 Old Main, University Park, PA 16802 USA

**Keywords:** PCR-based techniques, Genetic techniques

## Abstract

The fall armyworm (FAW), *Spodoptera frugiperda* (Smith), is a major pest native to the Americas. A recent invasion of FAWs from Africa eastward to South Asia, the Indochina Peninsula, and mainland China has received much attention due to the considerable economic losses in agriculture. FAWs can rapidly colonise a new area, likely due to the wide range of host plants, good flying capability, and high egg production. Therefore, a convenient, quick, and accurate tool for FAW identification is urgently required to establish a FAW invasion management strategy. In this study, FAW-specific primers were designed to recognise FAWs on the basis of internal transcribed spacer 1 (ITS1). The results revealed the accurate FAW recognition of the three congeneric species and eight common corn lepidopteran pests, especially at their larval stage. Furthermore, species-specific primers have confirmed their efficacy by using 69 FAW specimens from Taiwan, Thailand, and the United States, with a 96% success rate, excluding 3 decayed specimens. By using the simple, reliable, and convenient FAW-specific primers, a pest management programme can be developed not only to reduce sequencing costs and experimental time from 2 days to 4 h, but eradicate the FAW as soon as it enters a new area.

## Introduction

The fall armyworm (FAW), *Spodoptera frugiperda* (Smith) (Lepidoptera: Noctuidae), is a major pest native to the Americas with wide distribution from Canada to Argentina^1,2^. FAW is a highly polyphagous species that feeds on 76 families of host plants recorded in America, including more than 80 essential crops such as maize, sorghum, rice, cotton, alfalfa, and forage grasses^3,4^. Moreover, the FAW has a peculiar behaviour—it undergoes an annual long-distance migration, which has been proposed to be true for several lepidopteran taxa^1,2,5,6^. FAW adults migrate to southern Florida and Texas–Mexico in winter as they cannot survive the winter of Canada and the United States^1,7^; these two southern regions are also their native habitat. Thus, according to the biological features of FAWs, invasion events during a short interval are possible as well. Furthermore, the high volume of egg production by female FAWs—more than 1000 eggs during their lives—might be the factor for why FAW can colonise a new area and cause severe crop damage. Relevant reports concerning the severe economic losses caused by FAWs have been presented in various studies. For example, the damage caused by FAWs to economically essential crops such as maize, sorghum, rice, and sugarcane was estimated to be $13,383 million annually in Africa^8^.

The migratory capability and wide range of host plants increase the survival possibility of highly reproductive FAW when it colonises in a new area, either through natural migration or anthropogenic activities. In 2016, FAWs became notorious after their invasion of Nigeria and Ghana^9,10^ and then rapid intrusion, within 2 years, into > 20 sub-Saharan countries, causing severe economic losses of crops in Africa^11^. In 2018, FAW invaded Asian India in May, and thereafter, it swiftly migrated to Thailand, Sri Lanka, Bangladesh, Myanmar, Vietnam, and Laos^12–15^. In January 2019, FAW started to colonise Yunnan, China^14,16^, established populations, and then spread to nearly all southern Chinese provinces in a short time^17^. With the suspected assistance of southwestern monsoon, FAWs were found in west-central Taiwan in June 2019, despite Taiwan Island being isolated from the Asian mainland by Taiwan Strait, spanning a distance of more than 180 km. However, we could not exclude the artificial introduction of FAWs through human activities because many agricultural products are imported from Southeast Asia. Moreover, FAWs were detected in a maize field in southern Japan (Minamikyushu city, Kagoshima Prefecture) in July 2019 by the Plant Protection Station on 3 July 2019^18^. Indeed, Japan and Korea are at high risk of invasion^19,20^. The rapid population expansion of FAWs has received much attention because it leads to considerable economic losses. To prevent FAW invasion, a quick identification method is urgently required for FAW-free countries in Asia–Pacific regions, such as Japan, Korea, Australia, and New Zealand, and such a method is especially needed to identify FAW larvae.

The invasion events of FAW have also raised issues of the host strains of invasive populations. Genetic differentiation of FAWs with two known host strains—corn and rice—might have closely related to their larval host–plant adaptation^21^. However, recent molecular evidence has shown that both strains with undistinguishable morphology are generally sympatric in the field, implying the possibility of genetic exchange between the two evolutionary groups^22^. Moreover, evidence from amplified fragment length polymorphisms (AFLPs) has also revealed that two to five genetically distinct clusters existed in FAWs^22^; therefore, distinguishing the corn strain from rice strain based on their host plant is not feasible.

Taxonomic identification of FAW is generally difficult due to the co-occurrence of morphologically similar larvae of *Spodoptera* spp. in crops, although recognition of adult species is feasible. To date, the DNA barcodes using mitochondrial cytochrome oxidase subunit I (COI) have been used widely for species discrimination^9,10,22–26^. Furthermore, other amplicons used to identify FAW are the nuclear internal transcribed spacer 1 (ITS1) and triosephosphate isomerase^22–24^. Polymerase chain reaction (PCR)-based approaches such as restriction fragment length polymorphism and AFLPs have been used to analyse the population structure and identify the strain of FAW^22,27–29^. Although a draft genome assembly of FAWs was published by Kakumani et al.^30^, a convenient, quick, and easy molecular identification method for FAW is still undeveloped. Compared with DNA barcodes, specific primers can shorten the time of DNA product purification and sequencing for species identification from at least 2 days to 4 h. Thus, developing specific primers will be helpful to survey FAWs in pest-free countries because the transition of a FAW from larva to adult requires approximately 30 days^1,31^.

Among molecular amplicons used for rapid identification, the nuclear noncoding fragment, ITS1, with high interspecific genetic distance, has been widely used to develop species-specific primers in a single-step PCR for pest identification^32–35^. Lewter et al.^23^ found no variation in ITS1 for both strains of FAW, namely corn and rice strains, from several types of crops. The consistency of the ITS1 gene among populations was therefore helpful for divergent interspecific ITS1 to be used to design species-specific primers as a simple, quick, and reliable approach for identifying FAWs and distinguishing them from the other corn pests of *Spodoptera* spp.

This study aimed to identify FAWs on the basis of four pairs of species-specific primers designed using the ITS1 variable regions. In the field, identification of FAW larvae is generally difficult, especially that of young larvae, due to the co-existence of congeneric species. Thus, the detection of stable and efficacious FAW-specific primers for field specimens was performed for the following reasons: (1) to test the recognition ability among three common field congeneric species, (2) to examine the identification accuracy among field corn pests, and (3) to confirm the success rate of field FAW specimens.

## Results

### ITS1 sequence variations within and among *Spodoptera* species

Sequence variations of ITS1 were high among noctuid genera, ranging from 20.4% to 44.5% (Table 1). Gradual variations were generally found, and a saturation effect in a transversion substitution could be observed among divergent noctuid genera (see Supplementary Fig. S1c). Moreover, the twenty-eight ITS1 sequences of three *Spodoptera* species from GenBank, namely FAW, *S*. *litura*, and *S*. *exigua*, showed that sequence divergences were < 1.2% within species (Table 1). However, interspecific distance was much higher than intraspecific distances; FAW/*S*. *litura*, FAW/*S*. *exigua*, and *S*. *litura*/*S*. *exigua* were 16.2%, 19.3%, and 18.1%, respectively.

**Table 1:** Proportional divergences of the ITS1 gene among noctuid genera and *Spodoptera* species.

Genus	*Helicoverpa*	*Heliothis*	*Spodoptera*	*Mythimna*	*Trichoplusia*	*S*. *frugiperda*	*S*. *litura*	*S*. *exigua*	Species
*Helicoverpa*	–					1.2	16.2	19.3	*Sf*
*Heliothis*	20.4	–					0	18.1	*Sl*
*Spodoptera*	28.1	29.1	–					0.9	*Se*
*Mythimna*	36.4	36.0	37.7	–					
*Trichoplusia*	33.8	32.0	38.9	44.5	–				

### Stability of species-specific primers for FAW

**Figure 1:**
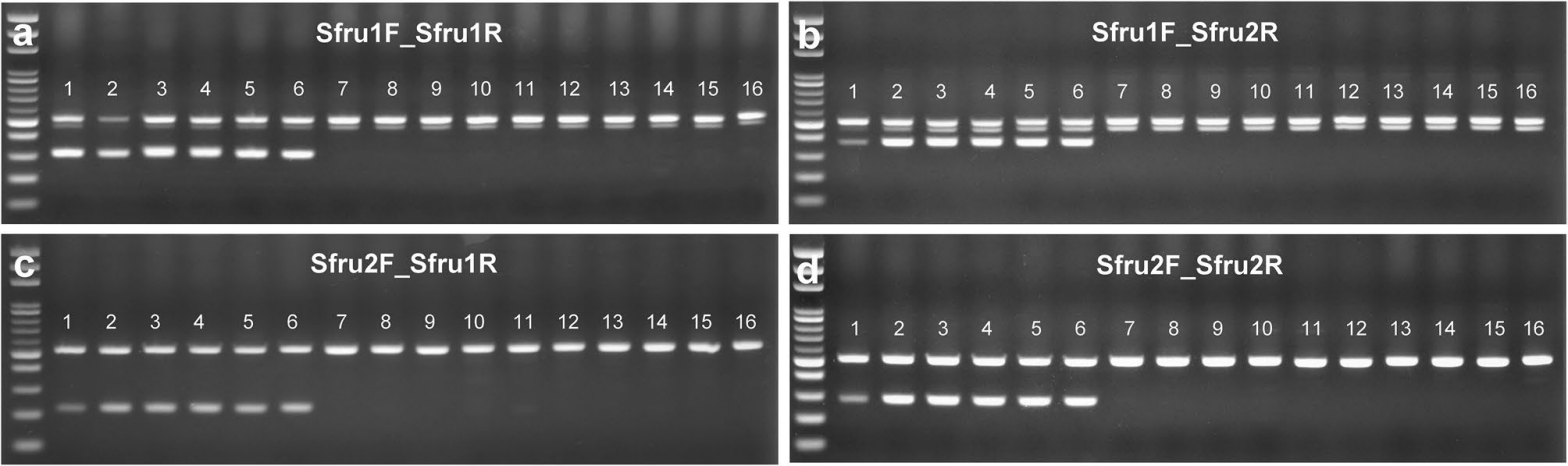
Multiplex PCR using a FAW-specific primer set with universal paired primers (cropped gel). A specific fragment is visible in target moths with no cross-amplification. The 100-bp DNA ladder and specific primer sets of Sfru1F_Sfru1R (**a**), Sfru1F_Sfru2R (**b**), Sfru2F_Sfru1R (**c**), and Sfru2F_Sfru2R (**d**) are shown on each panel. Lanes 1–6, FAW; 7–11, *Spodoptera litura*; and 12–16, *Spodoptera exigua*. Pertinent information of each individual of each lane is given in Supplementary Table S2. The uncropped images of gels are presented in Supplementary Figure S2.

**Figure 2:**
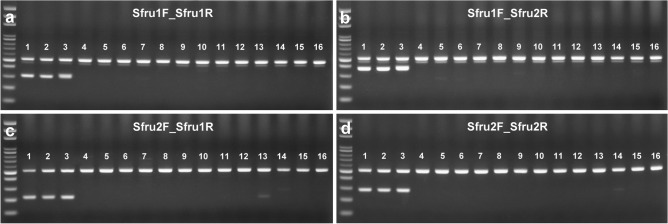
Stability test of a FAW-specific primer set with universal paired primers among the eight corn pests in the field (cropped gel). Specific amplification fragment is visible in target moths with no cross-amplification. The 100-bp DNA ladder and specific primer sets of Sfru1F_Sfru1R (**a**), Sfru1F_Sfru2R (**b**), Sfru2F_Sfru1R (**c**), and Sfru2F_Sfru2R (**d**) are shown on each panel. Lanes 1–3, FAW; 4 and 5, *Spodoptera exigua*; 6 and 7, *Euproctis taiwana*; 8 and 9, *Helicoverpa armigera*; 10, *Mythimna separate*; 11 and 12, *Mythimna loreyi*; 13 and 14, *Ostrinia furnacalis*; and 15 and 16, *Spoladea recurvalis*. Pertinent information of each individual of each lane is given in Supplementary Table S2. The uncropped images of gels are presented in Supplementary Figure S3.

The stability test of FAW-specific primers on the three *Spodoptera* species revealed that the expected amplified products were only amplified in target species, with no cross-amplification in the other two congeneric pests (Fig. 1). In each reaction, the successful amplification of universal primers of 28S rDNA with 520 bp confirmed the qualitative aspect of DNA and the experimental procedures. Additional weak bands were found with fragment sizes smaller than the universal primer products of Sfru1F/Sfru1R and Sfru1F/Sfru2R PCR amplifications, indicating no influence on the recognition of the specific products (Fig. 1a,b). For the eight common corn pests of noctuid moths, the stability of these species-specific primers showed that multiplex PCR can distinguish the FAW from other lepidopteran corn pests (Fig. 2). Although a weak band found in a nontarget species (Fig. 2c, lane 13: *O*. *furnacalis*) would somewhat lower the stability of the species identification, the relative DNA concentration without cross-amplification of other samples can eliminate this suspicion.

### Efficacy of specific primers for FAW identification

**Figure 3:**
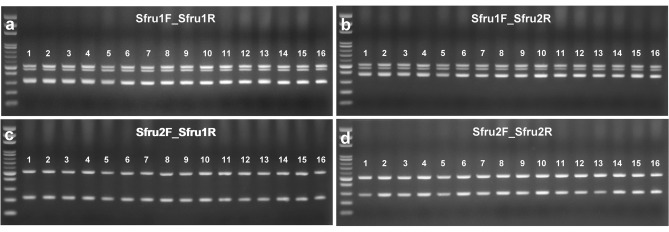
Efficacy test of a FAW-specific primer set with universal paired primers of partial FAW samples (cropped gel). The 100-bp DNA ladder and specific primer sets of Sfru1F_Sfru1R (**a**), Sfru1F_Sfru2R (**b**), Sfru2F_Sfru1R (**c**), and Sfru2F_Sfru2R (**d**) are shown on each panel. Pertinent information of each individual of each lane is given in Supplementary Table S2. The remaining FAW samples and uncropped images of gels are presented in Supplementary Figure S4.

Considering intraspecific variations of ITS1 in FAWs, the efficacy of four species-specific primers on 69 FAW specimens from Taiwan, Thailand, and the United States showed that specimens were amplified with a 96% success rate, except for three suspected decayed samples, which also led to failure or weak amplification in universal primers (Fig. 3, see Supplementary Fig. S4). However, their DNA concentrations ranged from 19.8 to 129.9 ng/μL did not show great difference from the other samples (see Supplementary Table S1). Furthermore, the dilution test of DNA concentration also revealed that the decreasing efficacy of FAW-specific primers occurred when DNA concentration was diluted to 100X and 1000X (see Supplementary Fig. S6).

### Application of specific primers for field FAW

**Figure 4:**
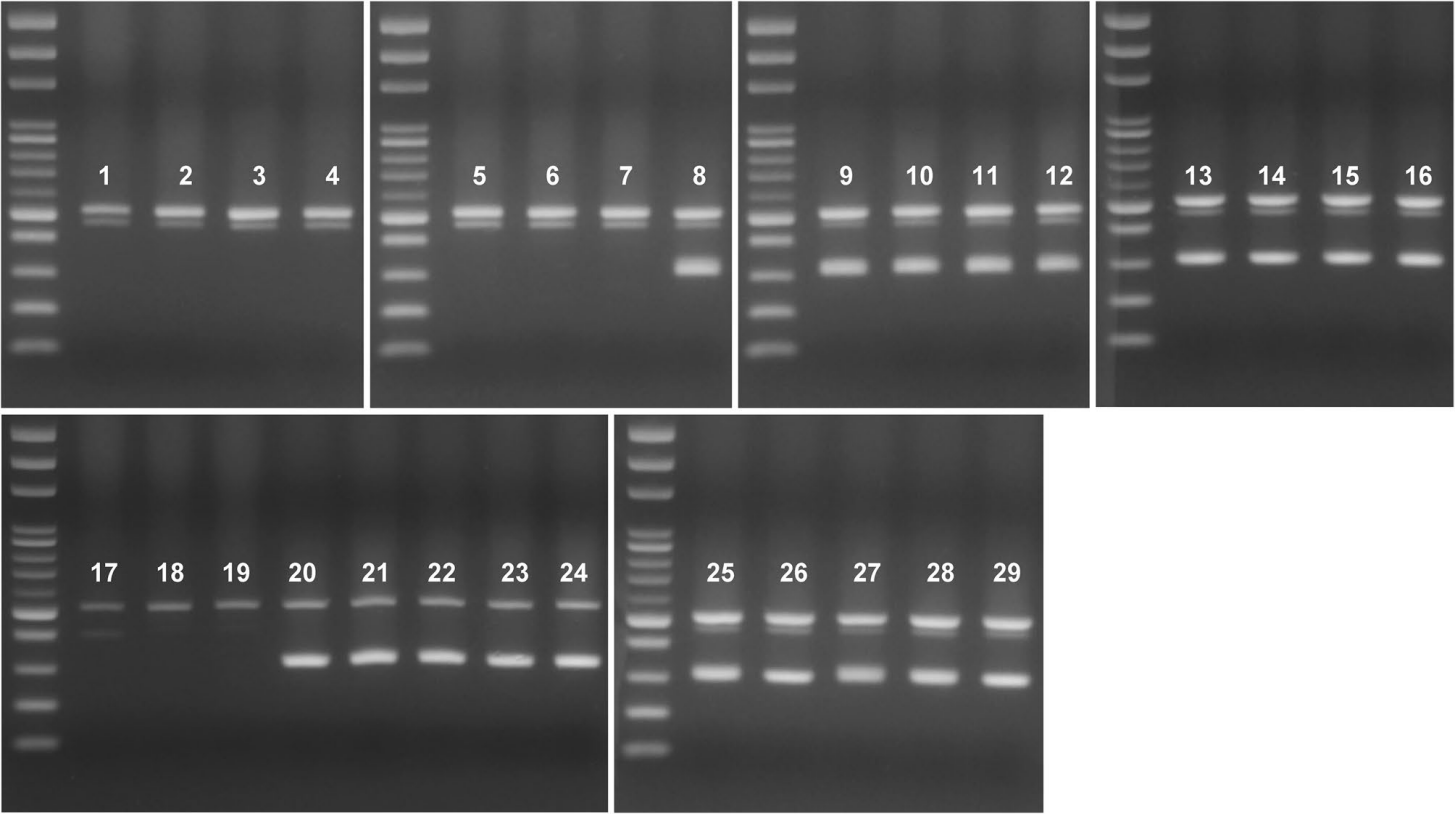
Six batches of field detection samples by using FAW-specific primer pair of Sfru1F/Sfru1R (cropped gel). The 100-bp DNA ladder is shown on the left lane. Pertinent information of each individual of each lane is given in Supplementary Table S2. The FAW samples tested by the other three FAW-specific primer pairs and the uncropped images of gels are presented in Supplementary Figure S5.

Specific primers were applied to identify larva samples of FAWs in corn fields. A total of six batches of larva samples were obtained from different corn fields, and each of them contained four to eight individuals. Resolutions showed that all four specific paired primers could be applied successfully to amplify FAW target fragments with no cross-amplification to other corn pests (Fig. 4, see Supplementary Fig. S5). One suspected decayed sample (lane 17) with a lower concentration of 28S rDNA amplification was observed (Fig. 4, see Supplementary Fig. S5). Moreover, COI sequences further confirmed the identity of these corn pests, namely *Hydrillodes lentalis*, *M*. *separate*, *M*. *loreyi*, *O*. *furnacalis*, and *Simplicia cornicalis* (Accession No. LC508666-LC508705) (see Supplementary Table S2).

## Discussion

Among the PCR-based approaches for species identification, multiplex PCR using universal primers, namely 28S rDNA, has ensured the DNA quality of sampled individuals^35,36^, which was seldom used. In this study, a few specimens died and rotted rapidly during their delivery process to our laboratory. Universal primers were useful to confirm the DNA quality for these decayed specimens in PCR experiments. Although the DNA concentration of the decayed samples showed no great difference to other samples, DNA fragmentation may be the possible explanation for their present status of failed amplification (see Supplementary Table S1 and Supplementary Fig. S6). Similarly, the DNA concentration dilution tests, which mimicked the DNA amounts of different field specimens, also affirmed that the failure of PCR amplification of 28S rDNA in these specimens was due to their spoiled status, particularly the DNA concentration diluted > 100X. In addition, the advantages of multiplex PCR can reduce the sequencing costs and time required for purification of DNA products from at least 2 days to 4 h, provided the experimental equipment is available. The identified results can provide the FAW management faculties with information for controlling this pest immediately as they collect the specimen in the field, especially in FAW-free countries.

Although the species-specific primers using mitochondrial genes are common in insects^37–39^, the more efficient nuclear ITS region with hypervariable sites, particularly the distinct inter- and intraspecific genetic variations of ITS sequences, is more suitable for developing species-specific primers for species identification^32,34–36,40–42^. However, the application of species-specific primers based on ITS sequences in insect identification remains rare. A few studies of mosquitos, thrips, and stored-product psocids have been proposed using ITS species-specific primers^35,36,41,43–46^. The results of the present study suggest that the ITS1 sequence variations of *Spodoptera* with high interspecific variations of > 16% and low intraspecific divergence of < 1.2% (Table 1) can be favourable markers with which to develop species-specific primers for FAW identification. The FAW-specific primers have accurately identified the FAW specimens from congeneric species and the other common lepidopteran pests in the field.

Doubts concerning the stability and efficacy of specific primers for FAW identification are raised as many noctuid moths or FAW populations are involved. For example, previous studies on thrips have shown that the detection ability of specific primers might fail if the number of mismatched positions of primers is more than four, but only one or two sporadically variable positions have little influence^41^. In the case of FAWs, Lewter et al.^23^ showed that no variation in ITS1 could be detected in samples belonging to both corn and rice strains from several types of crops in its native countries, namely the United States. Additional information from the nuclear ITS1 gene also revealed that gene flow may frequently occur in these two genetically differentiated strains, namely only 0.66% to 0.99% of the mitochondrial genes^23^. The slight genetic variation showed that these two host-specific strains may represent a recently diverged evolutionary units. Because no variation of the two strains was found in the ITS1 gene from its native habitat, the ITS1 of FAWs may have accumulated few or no mutations in the invasive populations within the past 5 years. Thus, the consistency of ITS1 among populations helped confirm the efficacy of the developed specific primer for FAWs. If the multiple invasion events occurred in the following years, the present species-specific primers should still work efficiently for FAW identification because nucleotide drift cannot occur in a short interval—several 1000 years. In addition, although a low concentration of the nontarget amplification products was observed in a specimen, which may have resulted from supercoiled DNA or a secondary structure of PCR product, the relatively high product yield of the target species could be applied concordantly for FAW identification (Fig. 2). The other three species-specific primer sets without PCR products amplification in the target position has eliminated this query. In this situation, the DNA barcodes are only required to confirm the identity of these two species, thus significantly elevating the efficiency of FAW identification.

An effective species recognition approach for FAW identification would be beneficial for establishing management strategies. On the basis of the biological habits of FAWs, FAW development from larva to adult requires approximately 30 days to establish its second generation in a new invasive area^1,31^. Because the damage related to FAW invasion predominantly occur in their larval stage, a rapid identification method is urgently required, especially considering that identifying the young larvae is more difficult than other larval instars and adults. Although pheromone traps are efficient for collecting adults for pest control, the FAW-invaded regions must take action at the initial invasion period to decrease its population establishment. Thus, it would be possible to eradicate FAWs as soon as it invades a new area if a quick identification method could be applied easily and conveniently. This study has displayed the efficiency of multiplex PCR using four pairs of specific primers in a systematic manner, which has also proved that these FAW-specific primers are simple, reliable, and convenient diagnostic tools for distinguishing FAW from other noctuid moths.

## Methods

### Sample collection

Fifty field larvae of FAW were collected from corn farms in Taiwan and Thailand (Taiwan: 31 specimens, including individuals of 2, 2, and 26 for 4th–6th instar larvae and 1 adult; Thailand: 19 individuals of 1, 11, 3, and 4 for 2nd, 4th–6th instar larvae; see Supplementary Table S2). Nineteen larvae specimens of a laboratory strain of FAW, including 2 eggs, individuals of 2, 4, 1, 1, 3, 2 for 1st–6th instar larvae, 3 pupae, and 1 destroyed specimen, were purchased from Benzon Research (Carlisle, PA, USA) (see Supplementary Table S2). The congeneric pests of *Spodoptera*, including *Spodoptera litura* (Fabricius) and *Spodoptera exigua* (Hübner), from Taiwan and Thailand as well as common corn pests in maize fields, namely *Euproctis taiwana* (Shiraki), *Helicoverpa armigera* (Hübner), *Mythimna separata* (Walker), *Mythimna loreyi* (Duponchel), *Ostrinia furnacalis* (Guenée), and *Spoladea recurvalis* (Fabricius), were used to test the stability of species-specific primers (for detailed information of instar stage and individuals, see Supplementary Table S2). The detailed information collected regarding the examined specimens is listed in Supplementary Table S2. All voucher specimens were preserved in 95% ethanol at − 20 °C and deposited in the Department of Entomology, National Chung Hsing University, Taichung, Taiwan.

### DNA extraction, amplification, and sequencing

Genomic DNA was extracted from adult/larval legs or eggs. In both adults and larvae, one leg was exploited for DNA extraction. The entire egg was used for DNA extraction. The tissue was ground in a 50-μL solution of the QuickExtract DNA extraction kit (Epicentre Biotechnologies, Madison, WI, USA) and was incubated at 65 °C for 10 min; it was then vortexed for 15 s followed by incubation at 98 °C for 2 min. After incubation, the sample solution was stored at − 20 °C for PCR. For each sample, the DNA concentration was measured using the Nanodrop 2000 Spectrophotometer (Thermo Scientific, Wilmington, DE, USA) before PCR amplification.

To confirm the identity of field larvae, the universal primer set of LCO1490 and HCO2198^47^ was used to amplify the mitochondrial COI gene, namely DNA barcodes, for species recognition. The PCR assay was conducted in 25 μL, including 19.8 μL of ddH_2_O, 2.5 μL of 10X GenTaq Buffer, 0.2 μL of 25 mM dNTP, 0.5 μL of both forward and reverse primers, 0.5 μL of GenTaq DNA polymerase (GenMark Technology, Taipei, Taiwan), and 1 μL of template DNA. The PCR conditions were as follows: incubation at 94 °C for 2 min as initial denaturation, 35 cycles of 94 °C for 40 s, 45 °C for 1 min, and 72 °C for 40 s, and then 72 °C for 10 min as a final extension. DNA products were checked by using 4.5 μL mixed with 1 μL of 6X EZ-Vision DNA Dye (Amresco Inc., Solon, OH, USA). Next, PCR products were run on a 1% agarose gel at 100 V. After electrophoresis, the gel was imaged using a Canon EOS M50 digital camera (Canon, LA, USA). A comparison based on DNA concentration of products and referable image (provided by Protech Technology Enterprise Co., Ltd., Taipei, Taiwan) was made to decide the DNA quality for sequencing. If the concentration of DNA products reached 109 ng, 20.5-μL samples of the remaining products were sent for sequencing. Samples were submitted to the two laboratories for sequencing, namely the Genome Research Center, Sequencing Center, National Yang Ming University and the Biotechnology Center, National Chung Hsing University. In the Genome Research Center, purification of PCR products was conducted using the QIAquick Gel Extraction Kit (Qiagen, Hilden, Germany) with 1% agarose gel. DNA products were sequenced using the BigDye Terminator 3.1 Sequencing Kit (Applied Biosystems, Foster City, CA, USA) and an ABI 3730XL sequencer. In the Biotechnology Center, DNA products were sequenced using an ABI 3730 DNA Analyzer.

### Sequence analyses of the noctuid ITS1 variation

ITS1 fragments of FAW (23 sequences), *S*. *litura* (2), *S*. *exigua* (3), and other noctuids (15) were retrieved from the GenBank (see Supplementary Table S2) and then aligned using Muscle Alignment option in SeaView4^48^. Genetic divergences among the noctuid moths were analysed on the basis of the ITS1 gene using MEGA 7.0 by assessing the p-distance^49^. Substitution saturation analysis was conducted through making a comparison of transitions and transversions versus the K80 distance using DAMBE v. 5.2.13^50^.

### Design of species-specific primers and multiplex PCR

Four pairs of species-specific primers of FAWs were designed on the basis of the variable regions of the ITS1 sequences of the three *Spodoptera* species, namely FAW, *S*. *litura*, and *S*. *exigua*, and noctuid moth sequences retrieved from GenBank. The stability of these species-specific primers was examined and determined among (1) the three *Spodoptera* pests and (2) the eight common corn pests, namely FAW, *S*. *exigua*, *E*. *taiwana*, *H*. *armigera*, *M*. *separata*, *M*. *loreyi*, *O*. *furnacalis*, and *Spoladea recurvalis*. Furthermore, the capability of species-specific primers was confirmed using 69 FAW specimens from Taiwan, Thailand, and the United States.

The upstream species-specific primers of FAW were Sfru1F (5ʹ-TTGCGCGAGACCGAGTG-3ʹ) and Sfru2F (5ʹ-TCTCGGACTTTAACACGT-3ʹ), and the downstream primers were Sfru1R (5ʹ-GCAATCGAAAAAGTTACAAAAAT-3ʹ) and Sfru2R (5ʹ-GGTAATAGTTTTAGATTCGTATC-3ʹ). The universal primers of upstream 28Sg (5ʹ-AGTTTGACTGGGGCGGTACA-3ʹ) and downstream 28Sh (5ʹ-CTTA GAGGCGTTCAGGCATAA-3ʹ)^51^ were used in each multiplex reaction as the qualitative control for confirming each experimental process. A multiplex PCR assay was performed in a total volume of 25 μL composed of 18.8 μL of ddH_2_O, 2.5 μL of 10X GenTaq Buffer, 0.2 μL of 25 mM dNTP, one specific primer set and the universal primers (0.5 μL per 10 μM primer), 0.5 μL of GenTaq DNA polymerase (GenMark, Technology, Taipei, Taiwan), and 1 μL of template DNA. Moreover, the template DNA of 16 samples was diluted to 10X, 100X, and 1000X, representing the different quality levels of field samples for sensitivity tests. The PCR programming conditions were as follows: denaturation at 94 °C for 2 min, followed by 30 cycles of denaturation at 94 °C for 20 s, annealing at 45 °C–51 °C for 40 s, and extension at 72 °C for 45 s, with a final extension at 72 °C for 10 min. The annealing temperatures of four specific primer sets were 46 °C, 48 °C, 50 °C, and 51 °C for Sfru2F/Sfru1R, Sfru2F/Sfru2R, Sfru1F/Sfru2R, Sfru1F/Sfru1R, respectively. DNA products were examined using 4.5 μL mixed with 1 μL of 6X EZ-Vision DNA Dye (Amresco Inc., Solon, OH, USA). Then, PCR products were run on a 2% agarose gel at 50 V. After electrophoresis, the gel was imaged using a Canon EOS M50 digital camera (Canon, LA, USA).

## Supplementary information

Supplementary Information 1.

## Data Availability

All sequences have been deposited in GenBank. Accession numbers and information of voucher specimens are listed in Supplementary Information.
