# Supplementary material for: Rapid identification of the invasive fall armyworm Spodoptera frugiperda (Lepidoptera, Noctuidae) using species-specific primers in multiplex PCR
Source: Sci Rep. 2020 Oct 5;10:16508. doi: 10.1038/s41598-020-73786-7 (PMC7536291; doi:10.1038/s41598-020-73786-7)

Rapid identification of the invasive fall armyworm *Spodoptera frugiperda* (Lepidoptera, Noctuidae) using species-specific primers in multiplex PCR

Cheng-Lung Tsai[1], I-Hsuan Chu[1], Ming-Hsun Chou[1], Theeraphap Chareonviriyaphap[2], Ming-Yao Chiang[3], Po-An Lin[4], Kuang-Hui Lu[1], Wen-Bin Yeh[1*]

[1]Department of Entomology, National Chung Hsing University; 145 Xingda Rd., South District, Taichung 40227, Taiwan.

[2]Department of Entomology, Faculty of Agriculture, Kasetsart University; 50 Ngamwongwan Rd., Chatuchak, Bangkok 10900, Thailand.

[3]Applied Zoology Division, Taiwan Agricultural Research Institute, Council of Agriculture, Executive Yuan; 189 Zhongzheng Rd., Wufeng District, Taichung 41326, Taiwan.

[4]Department of Entomology, Penn State University; 201 Old Main, University Park, Pennsylvania 16802, USA.

[*]Corresponding author, e-mail: wbyeh@nchu.edu.tw

Supplementary Table S1. Information on voucher ID and DNA concentration of each sample used in this study

Taxon	Voucher ID	DNA concentration (ng/µl)
Euproctis taiwana	Lep76-1	-
	Lep80-1	3.7
Helicoverpa armigera	Lep66-1	47.2
	Lep495-1	41.7
Hydrillodes lentalis	Lep580-3	96.3
Mythimna separata	Lep582-1	83.6
Mythimna loreyi	Lep582-2	73.1
	Lep582-3	173.3
Ostrinia furnacalis	Lep87-1	66.1
	Lep87-2	18.1
	Lep588-1	63.8
	Lep588-2	3.2
	Lep588-3	23.2
Simplicia cornicalis	Lep580-1	4.0
	Lep580-2	4.4
	Lep580-4	71.7
Spoladea recurvalis	Lep74-1	18.2
	Lep89-1	-
S. frugiperda	Lep581-2	54.8
	Lep581-3	218.6
	Lep581-4	25.8
	Lep581-5	24.6
	Lep581-6	11.6
	Lep581-7	23.2
	Lep581-8	32.2
	Lep581-9	26
	Lep581-10	78.2
	Lep581-11	65.4
	Lep581-12	86.1
	Lep581-13	147.6
	Lep581-14	19.8
	Lep581-15	52.8
	Lep581-16	50
	Lep581-17	47.9
	Lep581-18	101.8
	Lep581-19	48
	Lep581-20	90.4
	Lep582-4	110.3
	Lep583-1	85.7
	Lep583-2	91.2
	Lep583-3	89.5
	Lep583-4	26.8
	Lep584-1	57.1
	Lep584-2	56.2
	Lep584-3	102.9
	Lep584-4	87.6
	Lep589-1	157.2
	Lep590-1	120.4
	Lep590-2	68.6
	Lep591-1	61
	Lep591-2	79.9
	Lep592-1	62.7
	Lep592-2	68.3
	Lep592-3	113.1
	Lep592-4	82.5
	Lep592-5	61.5
	Lep652-1	90.4
	Lep652-2	117.1
	Lep670-1	30.6
	Lep670-2	65.9
	Lep670-3	62.5
	Lep682-1	15.3
	Lep683-1	129.9
	Lep683-2	105
	Lep683-3	171.6
	Lep683-4	31.6
	Lep683-5	100.3
	Lep683-6	140.8
	Lep752-1	135.8
	Lep752-2	141.8
	Lep752-3	62.1
	Lep752-4	57.6
	Lep752-5	62.7

Supplementary Table S1 (continued)

Taxon	Voucher ID	DNA concentration (ng/μl)
S. frugiperda	Lep752-6	45.9
	Lep752-7	31.6
	Lep752-8	21.5
	Lep752-9	26
	Lep752-10	34.4
	Lep752-11	43
	Lep752-12	25.8
	Lep752-13	45.9
	Lep752-14	58.6
	Lep752-15	21.4
	Lep752-16	22.8
	Lep752-17	46.2
	Lep752-18	24.2
	Lep752-19	52.3
S. litura	Lep65-1	-
	Lep65-2	72.8
	Lep191-1	69.1
	Lep528-1	96.5
	Lep535-1	39.9
S. exigua	Lep527-1	57.5
	Lep529-1	52.8
	Lep539-1	140.8
	Lep540-1	59.9
	Lep565-1	102.1

Supplementary Table S2. Information on voucher ID, instar stage, collecting locality, GPS coordinates, code in each figure, and accession numbers of noctuid moths. Sequences downloaded from GenBank are listed below the dashed line

Taxon	Voucher ID	Instar stage	Collecting locality	GPS-E	GPS-N	Code in this study									Accession No.
						Fig. 1	Fig. 2	Fig. 3	Fig. 4	Fig. S2	Fig. S3	Fig. S4	Fig. S5	Fig. S6	
Euproctis taiwana	Lep76-1	Adult	Fuchih Farm, Gukeng Township, Yunlin County, Taiwan	120.534811°	23.64085°		6				6				-
	Lep80-1	Adult	National Chiayi University, Taiwan	23.473190°	120.485723°		7				7				-
Helicoverpa armigera	Lep66-1	Unknown	National Chiayi University, Taiwan	23.473190°	120.485723°		8				8				-
	Lep495-1	3rd larva	Thailand	-	-		9				9				-
Hydrillodes lentalis	Lep580-3	4th larva	Fenyuan Township, Changhua County, Taiwan	-	-				3				3		LC508666
Mythimna separata	Lep582-1	5th larva	Wanggong, Fangyuan Township, Chuang County, Taiwan	23.961990°	120.338150°		10		5		10		5		LC508669
Mythimna loreyi	Lep582-2	5th larva	Wanggong, Fangyuan Township, Chuang County, Taiwan	23.961990°	120.338150°		11		6		11		6		LC508667
	Lep582-3	5th larva	Wanggong, Fangyuan Township, Chuang County, Taiwan	23.961990°	20.338150°		12		7		12		7		LC508668
Ostrinia furnacalis	Lep87-1	Adult	Fuchih Farm, Gukeng Township, Yunlin County, Taiwan	23.64085°	120.534811°		13				13				-
	Lep87-2	Adult	Fuchih Farm, Gukeng Township, Yunlin County, Taiwan	23.64085°	120.534811°		14				14				-
	Lep588-1	5th larva	Guoyesinduan, Husi Township, Penghu County, Taiwan	23.576453°	119.679679°				17				17		LC508670
	Lep588-2	5th larva	Guoyesinduan, Husi Township, Penghu County, Taiwan	23.576453°	119.679679°				18				18		LC508671
	Lep588-3	5th larva	Guoyesinduan, Husi Township, Penghu County, Taiwan	23.576453°	119.679679°				19				19		LC508672
Simplicia cornicalis	Lep580-1	5th larva	Fenyuan Township, Changhua County, Taiwan	-	-				1				1		LC508673
	Lep580-2	5th larva	Fenyuan Township, Changhua County, Taiwan	-	-				2				2		LC508674
	Lep580-4	5th larva	Fenyuan Township, Changhua County, Taiwan	-	-				4				4		LC508675
Spoladea recurvalis	Lep74-1	Adult	Fuchih Farm, Gukeng Township, Yunlin County, Taiwan	120.534811°	23.64085°		15				15				-
	Lep89-1	Adult	Fuchih Farm, Gukeng Township, Yunlin County	120.534811°	23.64085°		16				16				-
Spodoptera frugiperda	Lep581-2	5th larva	Department of Entomology, University of Pennsylvania, USA	-	-	1	1			1	1	17			LC508676
	Lep581-3	6th larva	Department of Entomology, University of Pennsylvania, USA	-	-	2	2			2	2	18			-
	Lep581-4	egg	Department of Entomology, University of Pennsylvania, USA	-	-							19		7	-
	Lep581-5	egg	Department of Entomology, University of Pennsylvania, USA	-	-							20		8	-
	Lep581-6	1st larva	Department of Entomology, University of Pennsylvania, USA	-	-							21		9	-
	Lep581-7	1st larva	Department of Entomology, University of Pennsylvania, USA	-	-							22		10	-
	Lep581-8	2nd larva	Department of Entomology, University of Pennsylvania, USA	-	-							23		11	-
	Lep581-9	2nd larva	Department of Entomology, University of Pennsylvania, USA	-	-							24			-
	Lep581-10	2nd larva	Department of Entomology, University of Pennsylvania, USA	-	-							25			-
	Lep581-11	2nd larva	Department of Entomology, University of Pennsylvania, USA	-	-							26			-
	Lep581-12	-	Department of Entomology, University of Pennsylvania, USA	-	-							27			-
	Lep581-13	3rd larva	Department of Entomology, University of Pennsylvania, USA	-	-							28			-
	Lep581-14	4th larva	Department of Entomology, University of Pennsylvania, USA	-	-							29			-
	Lep581-15	5th larva	Department of Entomology, University of Pennsylvania, USA	-	-							30			-
	Lep581-16	5th larva	Department of Entomology, University of Pennsylvania, USA	-	-							31			-
	Lep581-17	6th larva	Department of Entomology, University of Pennsylvania, USA	-	-							32			-
	Lep581-18	Pupa	Department of Entomology, University of Pennsylvania, USA	-	-							33			-
	Lep581-19	Pupa	Department of Entomology, University of Pennsylvania, USA	-	-							34			-
	Lep581-20	Pupa	Department of Entomology, University of Pennsylvania, USA	-	-							35			-
	Lep582-4	6th larva	Wanggong, Fangyuan Township, Chuang County, Taiwan	23.961990°	120.338150°			1	8			1	8		LC508677
	Lep583-1	6th larva	Cingshuei Township, Taichung City, Taiwan	-	-	3	3	2	9	3	3	2	9		-
	Lep583-2	6th larva	Cingshuei Township, Taichung City, Taiwan	-	-	4		3	10	4		3	10		LC508678
	Lep583-3	6th larva	Cingshuei Township, Taichung City, Taiwan	-	-			4	11			4	11		LC508679
	Lep583-4	5th larva	Cingshuei Township, Taichung City, Taiwan	-	-			5	12			5	12		LC508680
	Lep584-1	6th larva	Sihu Township, Changhua County, Taiwan	-	-	5		6	13	5		6	13		LC508681
	Lep584-2	5th larva	Sihu Township, Changhua County, Taiwan	-	-	6		7	14	6		7	14		LC508682

Supplementary Table S2 (continued)

Taxon	Voucher ID	Larval stage	Collecting Locality	GPS-E	GPS-N	Code in this study									Accession No.
						**Fig. 1**	**Fig. 2**	**Fig. 3**	**Fig. 4**	**Fig. S2**	**Fig. S3**	**Fig. S4**	**Fig. S5**	**Fig. S6**	
Spodoptera frugiperda	Lep584-3	6th larva	Sihu Township, Changhua County, Taiwan	-	-			8	15			8	15	1	LC508683
	Lep584-4	6th larva	Sihu Township, Changhua County, Taiwan	-	-			9	16			9	16	2	LC508684
	Lep589-1	6th larva	Guoyesinduan, Husi Township, Penghu County, Taiwan	23.576453°	119.679679°			10	20			10	20	3	LC508685
	Lep590-1	6th larva	Sisisinduan, Penghu County, Taiwan	23.581579°	119.638308°			11	21			11	21		LC508686
	Lep590-2	6th larva	Sisisinduan, Penghu County, Taiwan	23.581579°	119.638308°			12	22			12	22		LC508687
	Lep591-1	4th larva	Taiwu siduan, Penghu County, Taiwan	23.571097°	119.635640°			13	23			13	23		LC508688
	Lep591-2	Adult	Taiwu siduan, Penghu County, Taiwan	23.571097°	119.635640°			14	24			14	24		LC508689
	Lep592-1	6th larva	Checheng, Pingtung County, Taiwan	-	-			15	25			15	25	4	LC508690
	Lep592-2	6th larva	Checheng, Pingtung County, Taiwan	-	-			16	26			16	26		LC508691
	Lep592-3	6th larva	Checheng, Pingtung County, Taiwan	-	-				27			49	27		LC508692
	Lep592-4	6th larva	Checheng, Pingtung County, Taiwan	-	-				28			50	28		LC508693
	Lep592-5	6th larva	Checheng, Pingtung County, Taiwan	-	-				29			51	29		LC508694
	Lep652-1	6th larva	Houli District, Taichung City, Taiwan	24.315566°	120.715018°							52			LC508695
	Lep652-2	6th larva	Houli District, Taichung City, Taiwan	24.315566°	120.715018°							53			LC508696
	Lep670-1	6th larva	Mituo, Kaohsiung City, Taiwan	22.773713°	120.248315°							54			LC508697
	Lep670-2	6th larva	Mituo, Kaohsiung City, Taiwan	22.773713°	120.248315°							55			LC508698
	Lep670-3	6th larva	Mituo, Kaohsiung City, Taiwan	22.773713°	120.248315°							56			LC508699
	Lep682-1	6th larva	Luona, Sinyi Township, Nantou County, Taiwan	23.630372°	120.880075°							57			LC508700
	Lep683-1	4th larva	Annan District, Tainan City	23.071835°	120.190285°							58			-
	Lep683-2	6th larva	Annan District, Tainan City	23.071835°	120.190285°							59		5	LC508701
	Lep683-3	6th larva	Annan District, Tainan City	23.071835°	120.190285°							60		6	LC508702
	Lep683-4	6th larva	Annan District, Tainan City	23.071835°	120.190285°							61			LC508703
	Lep683-5	6th larva	Annan District, Tainan City	23.071835°	120.190285°							62			LC508704
	Lep683-6	6th larva	Annan District, Tainan City	23.071835°	120.190285°							63			LC508705
	Lep752-1	6th larva	Thailand	-	-							36		12	-
	Lep752-2	6th larva	Thailand	-	-							37		13	-
	Lep752-3	6th larva	Thailand	-	-							38		14	-
	Lep752-4	6th larva	Thailand	-	-							39		15	-
	Lep752-5	4th larva	Thailand	-	-							40		16	-
	Lep752-6	5th larva	Thailand	-	-							41			-
	Lep752-7	4th larva	Thailand	-	-							42			-
	Lep752-8	4th larva	Thailand	-	-							43			-
	Lep752-9	4th larva	Thailand	-	-							44			-
	Lep752-10	4th larva	Thailand	-	-							45			-
	Lep752-11	5th larva	Thailand	-	-							46			-
	Lep752-12	4th larva	Thailand	-	-							47			-
	Lep752-13	4th larva	Thailand	-	-							48			-
	Lep752-14	5th larva	Thailand	-	-							64			-
	Lep752-15	4th larva	Thailand	-	-							65			-
	Lep752-16	4th larva	Thailand	-	-							66			-
	Lep752-17	4th larva	Thailand	-	-							67			-
	Lep752-18	4th larva	Thailand	-	-							68			-
	Lep752-19	2nd larva	Thailand	-	-							69			-
S. litura	Lep65-1	Adult	National Chiayi University, Taiwan	23.473190°	120.485723°	7				7					-
	Lep65-2	Adult	National Chiayi University, Taiwan	23.473190°	120.485723°	8				8					-
	Lep191-1	Adult	Nanao Township, Yilan County, Taiwan	24.423041°	121.789871°	9				9					-

Supplementary Table S2 (continued)

Taxon	Voucher ID	Larval stage	Collecting Locality	GPS-E	GPS-N	Code in this study									Accession No.
						Fig. 1	Fig. 2	Fig. 3	Fig. 4	Fig. S2	Fig. S3	Fig. S4	Fig. S5	Fig. S6	
S. litura	Lep528-1	2nd larva	Thailand	-	-			10				10			-
	Lep535-1	1st larva	Thailand	-	-			11				11			-
S. exigua	Lep527-1	2nd larva	Thailand	-	-			12				12			-
	Lep529-1	2nd larva	Thailand	-	-			13	4			13			-
	Lep539-1	2nd larva	Thailand	-	-			14	5			14			-
	Lep540-1	3rd larva	Thailand	-	-			15				15			-
	Lep565-1	3rd larva	Thailand	-	-			16				16			-
Helicoverpa armigera	-	-	-	-	-	-	-	-	-	-	-	-	-	-	EU204143
	-	-	-	-	-	-	-	-	-	-	-	-	-	-	KT343377
	-	-	-	-	-	-	-	-	-	-	-	-	-	-	KT343378
	-	-	-	-	-	-	-	-	-	-	-	-	-	-	AJ577253
	-	-	-	-	-	-	-	-	-	-	-	-	-	-	AB620127
	-	-	-	-	-	-	-	-	-	-	-	-	-	-	AF401740
Helicoverpa zea	-	-	-	-	-	-	-	-	-	-	-	-	-	-	KT343375
	-	-	-	-	-	-	-	-	-	-	-	-	-	-	KT343376
	-	-	-	-	-	-	-	-	-	-	-	-	-	-	KT343380
	-	-	-	-	-	-	-	-	-	-	-	-	-	-	KT343381
Helicoverpa assulta	-	-	-	-	-	-	-	-	-	-	-	-	-	-	KT343382
Heliothis subflexa	-	-	-	-	-	-	-	-	-	-	-	-	-	-	KT762150
Heliothis virescens	-	-	-	-	-	-	-	-	-	-	-	-	-	-	KT343379
Mythimna separata	-	-	-	-	-	-	-	-	-	-	-	-	-	-	LC361446
S. exigua	-	-	-	-	-	-	-	-	-	-	-	-	-	-	JN863291
	-	-	-	-	-	-	-	-	-	-	-	-	-	-	JN863292
	-	-	-	-	-	-	-	-	-	-	-	-	-	-	JN863293
S. litura	-	-	-	-	-	-	-	-	-	-	-	-	-	-	FJ041111
	-	-	-	-	-	-	-	-	-	-	-	-	-	-	JN863294
S. frugiperda	-	-	-	-	-	-	-	-	-	-	-	-	-	-	GQ478352
	-	-	-	-	-	-	-	-	-	-	-	-	-	-	MH779574
	-	-	-	-	-	-	-	-	-	-	-	-	-	-	MH779575
	-	-	-	-	-	-	-	-	-	-	-	-	-	-	MH779576
	-	-	-	-	-	-	-	-	-	-	-	-	-	-	MH779577
	-	-	-	-	-	-	-	-	-	-	-	-	-	-	MH779578
	-	-	-	-	-	-	-	-	-	-	-	-	-	-	MH779579
	-	-	-	-	-	-	-	-	-	-	-	-	-	-	MH779580
	-	-	-	-	-	-	-	-	-	-	-	-	-	-	MH779581
	-	-	-	-	-	-	-	-	-	-	-	-	-	-	MH779582
	-	-	-	-	-	-	-	-	-	-	-	-	-	-	MH779583
	-	-	-	-	-	-	-	-	-	-	-	-	-	-	MH779584
	-	-	-	-	-	-	-	-	-	-	-	-	-	-	MH779585
	-	-	-	-	-	-	-	-	-	-	-	-	-	-	MH819372
	-	-	-	-	-	-	-	-	-	-	-	-	-	-	MH819373
	-	-	-	-	-	-	-	-	-	-	-	-	-	-	MH819374
	-	-	-	-	-	-	-	-	-	-	-	-	-	-	MH819375
	-	-	-	-	-	-	-	-	-	-	-	-	-	-	MH819376
	-	-	-	-	-	-	-	-	-	-	-	-	-	-	MH819377
	-	-	-	-	-	-	-	-	-	-	-	-	-	-	MH819378

Supplementary Table S2 (continued)

Taxon	Voucher ID	Larval stage	Collecting Locality	GPS-E	GPS-N	Code in this study									Accession No.
						Fig. 1	Fig. 2	Fig. 3	Fig. 4	Fig. S2	Fig. S3	Fig. S4	Fig. S5	Fig. S6	
S. frugiperda	-	-	-	-	-	-	-	-	-	-	-	-	-	-	MH819379
	-	-	-	-	-	-	-	-	-	-	-	-	-	-	MH819380
	-	-	-	-	-	-	-	-	-	-	-	-	-	-	MH819381
	-	-	-	-	-	-	-	-	-	-	-	-	-	-	HM851175

Supplementary Figure S1. Plot distribution of ITS1 sequence variations in categories of genus *Helicoverpa* **(a)**, genus *Spodoptera* **(b)**, and Noctuidae **(c)**.

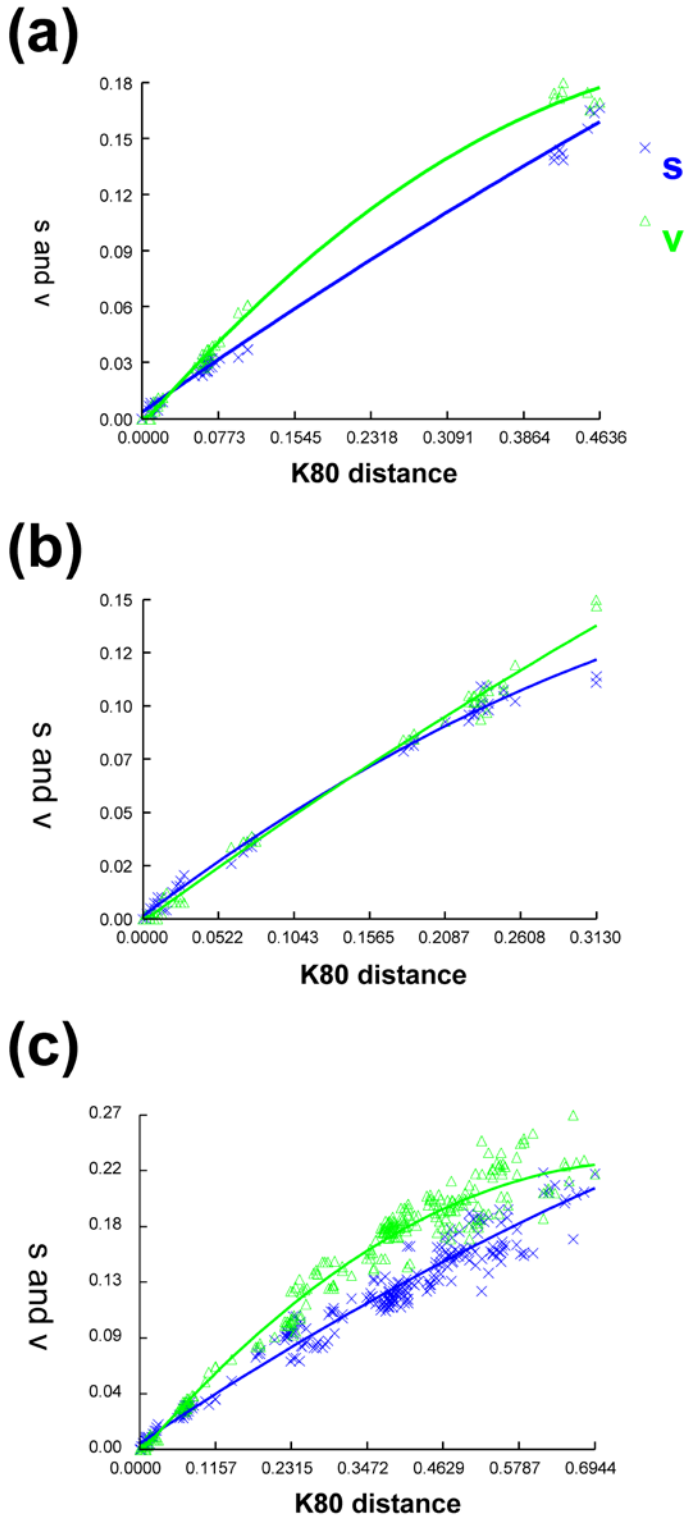

Supplementary Figure S2. Multiplex PCR using a FAW-specific primer set with universal paired primers (uncropped gel). A specific fragment is visible in target moths with no cross-amplification. The 100-bp DNA ladder and specific primer sets of Sfru1F_Sfru1R **(a)**, Sfru1F_Sfru2R **(b)**, Sfru2F_Sfru1R **(c)**, and Sfru2F_Sfru2R **(d)** are displayed on each panel. Lanes 1–6, FAW; 7–11, *Spodoptera litura*; and 12–16, *Spodoptera exigua*. Pertinent information of each individual of each lane is presented in **Supplementary Table S2**.

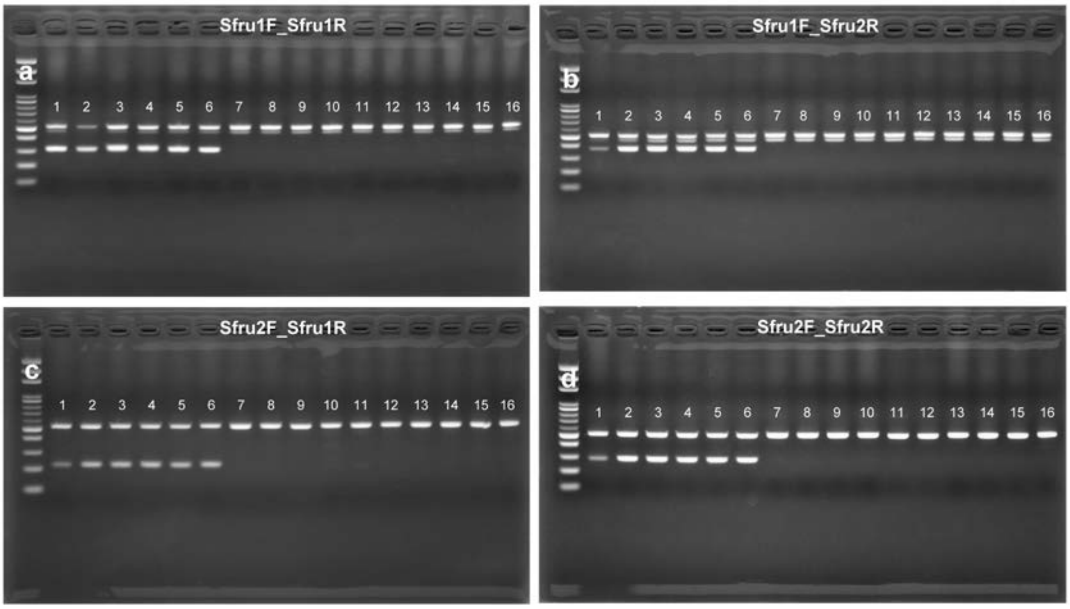

Supplementary Figure S3. Stability test of a FAW-specific primer set with universal paired primers among the eight corn pests in the field (uncropped gel). Specific amplification fragment is visible in target moths with no cross-amplification. The 100-bp DNA ladder and specific primer sets of Sfru1F_Sfru1R **(a)**, Sfru1F_Sfru2R **(b)**, Sfru2F_Sfru1R **(c)**, and Sfru2F_Sfru2R **(d)** are displayed on each panel. Lanes 1–3, FAW; 4 and 5, *Spodoptera exigua*; 6 and 7, *Euproctis taiwana*; 8 and 9, *Helicoverpa armigera*; 10, *Mythimna separate*; 11 and 12, *Mythimna loreyi*; 13 and 14, *Ostrinia furnacalis*; and 15 and 16, *Spoladea recurvalis*. Pertinent information of each individual of each lane is given in **Supplementary Table S2**.

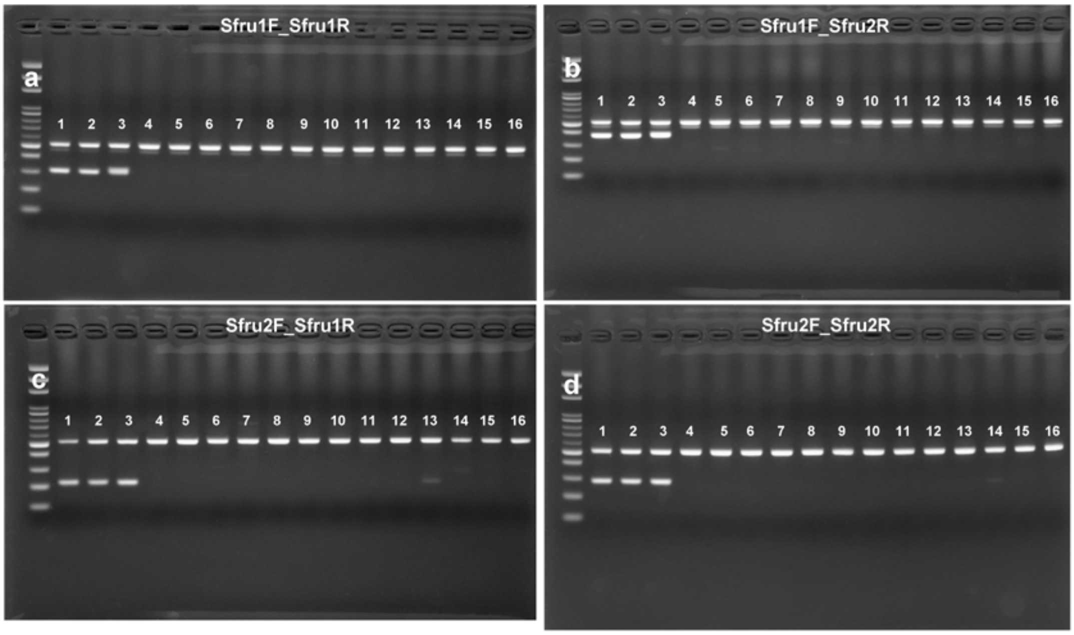

Supplementary Figure S4. Efficacy test of a FAW-specific primer set with universal paired primers of partial FAW samples (uncropped gel). The 100-bp DNA ladder and specific primer sets of Sfru1F_Sfru1R **(a—e)**, Sfru1F_Sfru2R **(f—j)**, Sfru2F_Sfru1R **(k—o)**, and Sfru2F_Sfru2R **(p—t)**, are displayed on each panel. Pertinent information on each individual of each lane is presented in **Supplementary Table S2**.

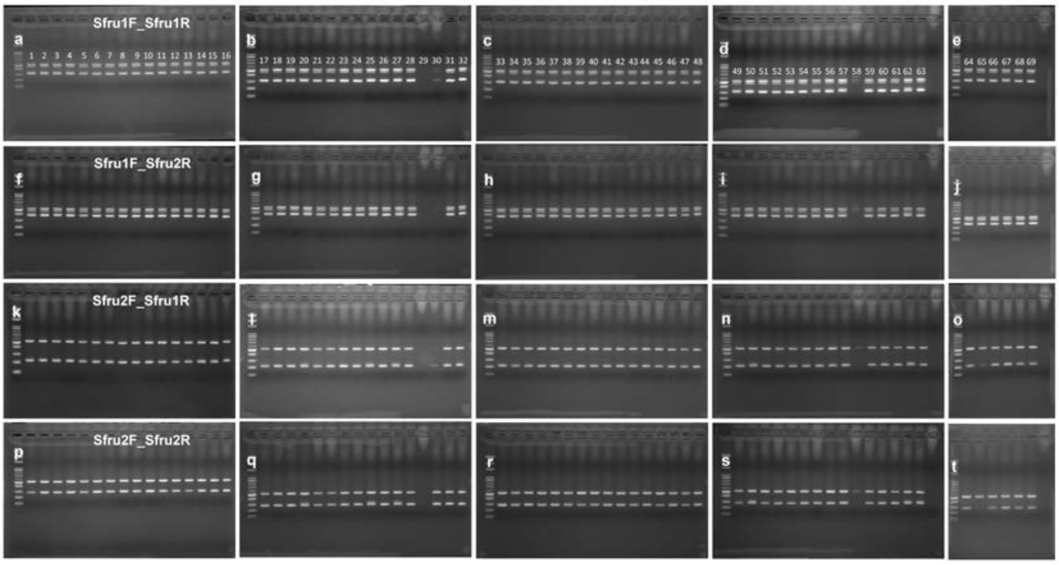

Supplementary Figure S5. Field sample detection by using FAW-specific primer pairs of Sfru1F_Sfru1R, Sfru1F_Sfru2R, Sfru2F_Sfru1R, and Sfru2F_Sfru2R (uncropped gel). The 100-bp DNA ladder is shown on the left lane. Pertinent information of each individual of each lane is presented in **Supplementary Table S2**.

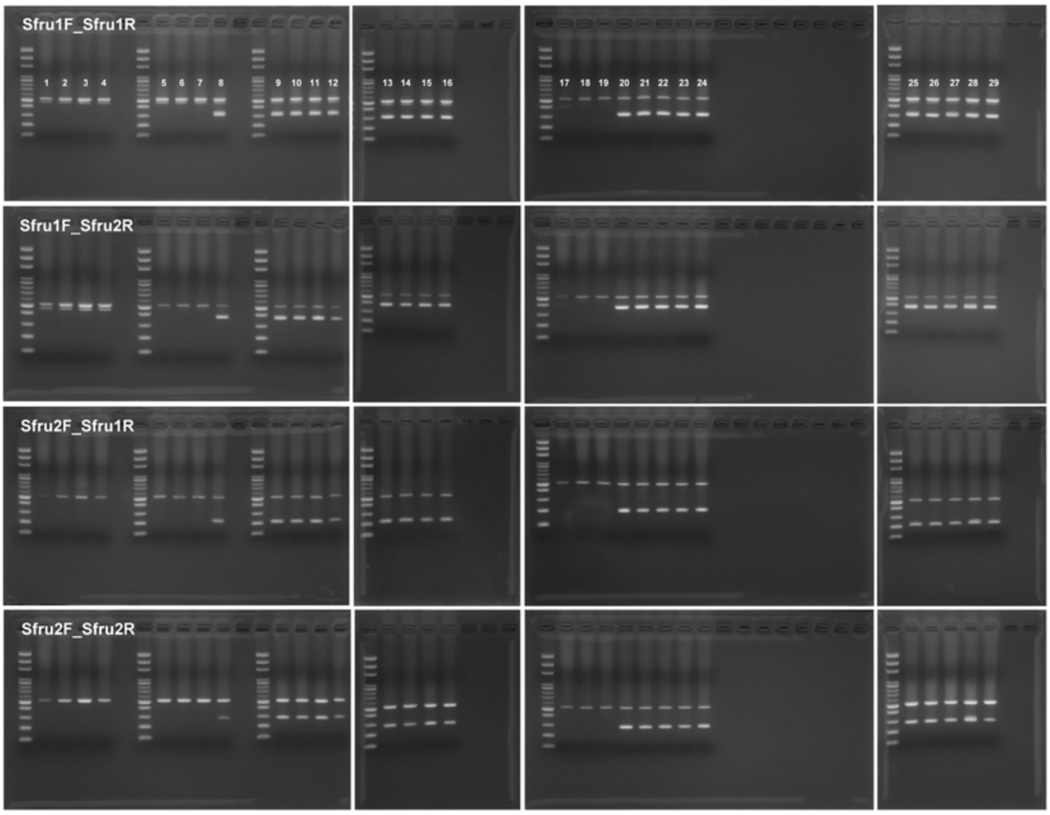

Supplementary Figure S6. FAWs DNA dilution test of 1X (**a**—**d**), 10X (**e**—**h**), 100X (**i** — **l**), and 1,000X (**m** — **p**) using FAW-specific primer pairs of Sfru1F_Sfru1R, Sfru1F_Sfru2R, Sfru2F_Sfru1R, and Sfru2F_Sfru2R (uncropped gel). The 100-bp DNA ladder is shown. Pertinent information of each individual of each lane is presented in **Supplementary Table S2**.

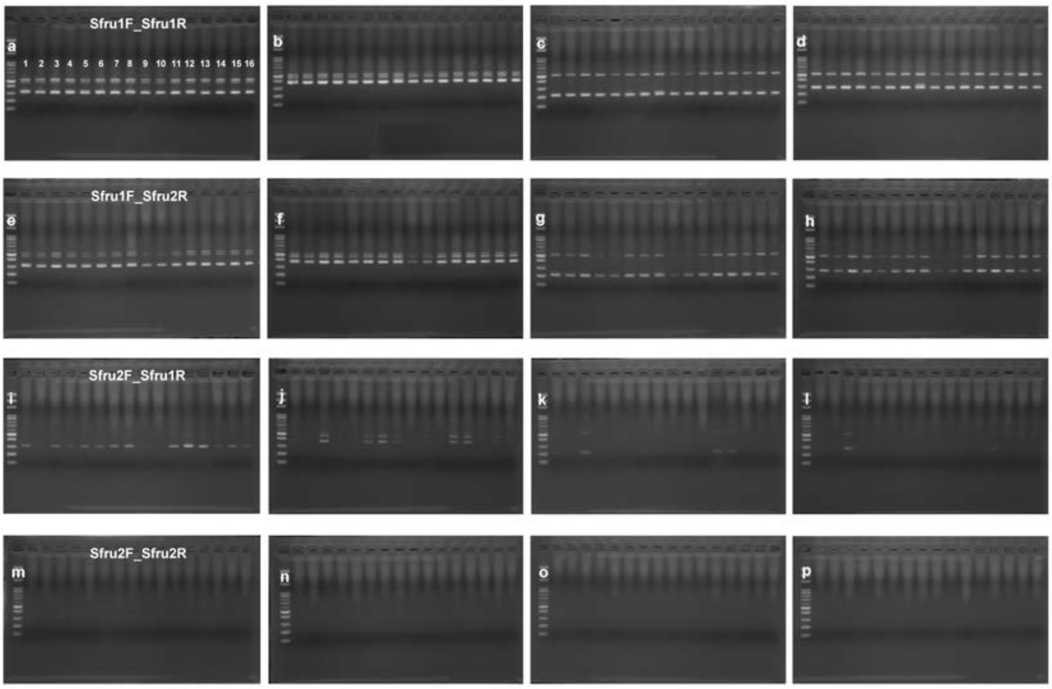

Supplement: Supplementary file 1 — Supplementary Information 1. [file 41598_2020_73786_MOESM1_ESM.pdf]
